# Tgf-β1 transcriptionally promotes 90K expression: possible implications for cancer progression

**DOI:** 10.1038/s41420-021-00469-1

**Published:** 2021-04-22

**Authors:** Antonino Grassadonia, Vincenzo Graziano, Sara Pagotto, Angelo Veronese, Cesidio Giuliani, Marco Marchisio, Paola Lanuti, Michele De Tursi, Maurizia D’Egidio, Pietro Di Marino, Davide Brocco, Patrizia Vici, Laura De Lellis, Alessandro Cama, Clara Natoli, Nicola Tinari

**Affiliations:** 1 Department of Medical, Oral and Biotechnological Sciences and Center for Advanced Studies and Technology (CAST), G. D’Annunzio University, Chieti, Italy; 2 Cancer Research UK Cambridge Institute, University of Cambridge, Cambridge, UK; 3 Department of Medicine and Ageing Sciences and Center for Advanced Studies and Technology (CAST), G. D’Annunzio University, Chieti, Italy; 4 Division of Medical Oncology 2, IRCCS Regina Elena National Cancer Institute, Rome, Italy; 5 Department of Pharmacy and Center for Advanced Studies and Technology (CAST), G. D’Annunzio University, Chieti, Italy

**Keywords:** Growth factor signalling, Transcriptional regulatory elements

## Abstract

The 90K protein, also known as Mac-2 BP or LGALS3BP, can activate the immune response in part by increasing major histocompatibility (MHC) class I levels. In studies on a non-immune cell model, the rat FRTL-5 cell line, we observed that transforming growth factor (TGF)-β1, like γ-interferon (IFN), increased 90K levels, despite its immunosuppressive functions and the ability to decrease MHC class I. To explain this paradoxical result, we investigated the mechanisms involved in the TGF-β1 regulation of 90K expression with the aim to demonstrate that TGF-β1 utilizes different molecular pathways to regulate the two genes. We found that TGF-β1 was able to increase the binding of Upstream Stimulatory Factors, USF1 and USF2, to an E-box element, CANNTG, at −1926 to −1921 bp, upstream of the interferon response element (IRE) in the 90K promoter. Thyrotropin (TSH) suppressed constitutive and γ-IFN-induced 90K expression by decreasing USF binding to the E-box. TGF-β1 was able to overcome TSH suppression at the transcriptional level by increasing USF binding to the E-box. We suggest that the ability of TGF-β1 to increase 90K did not result in an increase in MHC class I because of a separate suppressive action of TGF-β1 directly on the MHC class I gene. We propose that the increased levels of 90K may play a role, rather than in immune response, in the context of the TGF-β1-induced changing of the cellular microenvironment that predisposes to cell motility and cancer progression. Consistently, analyzing the publicly available cancer patient data sets cBioPortal, we found that 90K expression directly correlated with TGF-β1 and USFs and that high levels of 90K were significantly associated with increased mortality in patients affected by different types of cancer.

## Introduction

The Transforming growth factor-beta (TGF-β) polypeptides regulate the growth, function, and immune properties of cells^1^. The prevalent role of TGF-β1 in the immune system is to induce tolerance and immune suppression^2^, in part by inhibiting IL-2 production and NK activity^3,4^, promoting the differentiation of naive T-cells into Treg via Foxp3 transcription factor^5,6^, and decreasing the expression of major histocompatibility complex (MHC) class I^7^. The importance of suppressing class I levels is clear since TGF-β1-deficient transgenic mice have increased MHC class I levels in many organs and develop a rapid, wasting, autoimmune disease^8^.

The 90K protein, also known as Mac-2 BP or LGALS3BP, is an oligomeric glycoprotein that has been identified in the serum of patients with tumors or acquired immunodeficiency syndrome (AIDS)^9^. In contrast to TGF-β1, 90K activates the immune defense systems of the organism^10^. In particular, human 90K enhances the in vitro generation of cytotoxic effector cells (NK and LAK) from peripheral blood mononuclear cells (PBMC)^11^; increases IL-2 production by PBMC^11^ and increases MHC class I antigen expression in human breast cancer cells^12^.

To better understand the physiological role of 90K, we have characterized its function in rat FRTL-5 thyroid cells. These cells were chosen because they can maintain a normal functioning behavior in culture: (i) near diploid, (ii) absence of tumor phenotype, (iii) growth and immune-related protein expression under the control of TSH^13^. In FRTL-5 cells, we have previously reported that rat 90K, similarly to its human homologous, was increased by γ-IFN and that TSH was able to decrease both constitutive and γ-IFN-induced 90K synthesis^14^. We have also shown that rat 90K acted in an autocrine or paracrine/exocrine manner to increase MHC class I and that it was a component of an immune response aimed at converting non-immune cells to potential antigen-presenting cells (APCs)^14^. This effect was triggered by the presence of foreign double-strand nucleic acids in the cytoplasm (viral, bacterial, or self)^14^.

As expected, in rat FRTL-5 thyroid cells TGF-β1 decreases expression of MHC class I^15^ and inhibits cell proliferation as well as the thyrotropin (TSH)-induced functions such as iodide uptake and thyroglobulin production^16,17^.

Thus, given the antagonistic properties of TGF-β1 and 90K within the immune system and their opposite effects on MHC class I regulation in FRTL-5 cells, we investigated the effect of TGF-β1 on the expression of 90K. Surprisingly, we found that TGF-β1-increased 90K gene expression.

In the present study, we investigated the mechanism by which this occurred in the attempt to explain the apparent paradox and better clarify the biologic function of 90K.

## Material and methods

### Cells

Rat FRTL-5 thyroid cells (Interthyr Corporation, Marietta, OH, USA) were a fresh subclone (F1) with all properties described^18^. They were grown in 6H medium consisting of Coon’s modified F12 medium, 5% heat-treated, mycoplasma-free, calf serum, 1 mM nonessential amino acids, and a six hormone mixture: bovine TSH (1 × 10^−10^M), insulin (10 μg/ml), cortisol (0.4 ng/ml), transferrin (5 μg/ml), glycyl-l-histidyl-l-lysine acetate (10 ng/ml), and somatostatin (10 ng/ml). Cells were fed every 2–3 d, were passaged every 7–10 d, were diploid and were between their fifth and 25th passage. Their doubling time with TSH was 36 ± 6 h; without TSH, they did not proliferate nor exhibit most thyroid-specific functions. As noted, in some experiments cells were maintained in 5H medium (no TSH) then stimulated with 1 × 10^−10^ M TSH, 100 U/ml rat γ-IFN, or 5 ng/ml TGF-β1 for 24 h unless otherwise noted. Experiments carried out using forskolin (5 μM) and IGF-I (100 ng/ml) instead of TSH and insulin, respectively, showed similar results.

U2OS cells (ATCC^®^ HTB-96^TM^), a human osteosarcoma cell line suitable for co-transfection experiments, were grown in Dulbecco’s Modified Eagle’s Medium with 4.5 g/l glucose, supplemented with 10% fetal bovine serum, 1% Pen/Strep and 1% l-glutamine (Merck, Darmstadt, Germany).

### RNA isolation, northern blot analysis, and RT-qPCR

For northern blot, RNA was isolated using the commercial kit Quickprep mRNA Purification (Pharmacia Biotech, Uppsala, Sweden) and northern analysis was performed using Nytran nitrocellulose membranes (Merck, Darmstadt, Germany) as described^14^. Filters were hybridized with the rat 90K and GAPDH cDNAs; radiolabeling of probes and hybridization (1 × 10^6^ cpm/ml) were also as described^14^. Rat 90K cDNA was the full-length clone previously described^14^.

For reverse transcription-quantitative PCR (RT-qPCR), total RNA was isolated using QIAzol Lysis Reagent (Qiagen, Hilden, Germany) according to the manufacturer’s instruction and reverse-transcribed with the High Capacity cDNA Reverse Transcription Kit (ThermoFisher Scientific, Waltham, Massachusetts, USA). RT-qPCR was performed using the QuantiFast SYBR® Green PCR Kit (Qiagen, Hilden, Germany) using specific rat 90K primers, F: 5′-CTGAACAGTCTACAGAAAGCTTCG-3′ and R: 5′-GACCTGGAAGCCCAACCT-3′; and GAPDH primers, F: 5′-TGGGAAGCTGGTCATCAAC-3′ and R: 5′-GCATCACCCCATTTGATGTT-3′. Melting curve analyses were performed according to the specification of the CFX96 Touch™ Real-Time PCR Detection System (Bio-Rad, Hercules, CA, USA). 90K mRNA expression was normalized to the endogenous reference GAPDH using the 2-Δct method (User Bulletin #2, ThermoFisher Scientific, Waltham, Massachusetts, USA).

### In vivo labeling and immunoprecipitation

FRLT-5 cells in 5H medium were stimulated with 1 × 10^−10^ M TSH, 100 U/ml rat γ-IFN, or 5 ng/ml TGF-β1 as noted above. Biosynthetic labeling of proteins was performed during the last 12 h by maintaining the cells overnight with ^35^S-methionine (50 μCi/ml) in methionine-free F12 Coon’s modified medium (0.5 ml/well) containing 1% dialyzed FCS (ThermoFisher Scientific, Waltham, MA, USA). The spent medium was precleared with rabbit preimmune serum and incubated with the polyclonal anti-90K antibody^14^, followed by protein A-Sepharose (Merck, Darmstadt, Germany). The immunoprecipitate was separated by SDS-PAGE and proteins detected by autoradiography.

### Oligonucleotides and plasmids

USF expression plasmids (pUSF1 and pUSF2) were generated by cloning USF1 or USF2 DNA into pCMV-tag2A vectors (Agilent Technologies, Santa Clara, CA, USA), as previously described^19^. The rat 90K promoter-luciferase chimera, containing the fragment spanning position −1963 to −1772 bp of the 90K 5′-flanking region subcloned in a pGL3 basic vector (Promega, Madison, WI, USA), was also described^14^. This region referred to as P2, corresponds to the minimal promoter defined by sequence homology among rat, human, and mouse genes and is functionally relevant as it contains the interferon and TSH response elements^20^. Using site-specific PCR mutagenesis procedure, we created a P2 fragment with four base substitutions in the interferon response element (IRE) (−1912 to −1902 bp). This construct, indicated as P2IREM, has the IRE sequence changed in **tc**A **g**C**t** GAA GCT and was also subcloned in a pGL3 basic vector (Promega, Madison, WI, USA).

Oligo I–II is the region spanning from −1963 to −1912 bp in the rat 90K minimal promoter; Oligo II is from −1963 to −1932: 5′-AGC CTT GTC TGC AGC CAA CCC CAG AGG CAG CC-3′; Oligo I is from −1937 to −1912 bp: 5′-GCA GCC TCC GTC ATG TGT TTT CTG GA-3′. CM3, CM2, CM1, TM1, TM2, and CEM are two-base substitutions of Oligo I: CM3, 5′-GCA **a**C**a** TCC GTC ATG TGT TTT CTG GA-3′; CM2, 5′-GCA GC**a** TC**a** GTC ATG TGT TTT CTG GA-3′; CM1, 5′-GCA GCC TC**a** G**g**C ATG TGT TTT CTG GA-3′; TM1, 5′-GCA GCC TCC GTC AT**c** TGT TT**c** CTG GA-3′; TM2, 5′-GCA GCC TCC GTC ATG TGT TT**c a**TG GA-3′; and CEM, 5′-GCA GCC TCC GTC ATG **ga**T TTT CTG GA-3′. Lowercase and bold letters denote the substituted nucleotides. The oligonucleotide containing the consensus E-box binding site (bold and underlined), indicated as E, is 5′-GGA AGC AGA C**CA CGT G**GT CTG CTT-3; EM is a two-base mutation (lowercase) in the E-box of E, 5′-GGA AGC AGA C**CA CGg a**GT CTG CTT-3′. The cAMP response element (CRE) competitor was 5′-AGA GAT CTG ACG TCA GAC AGC TAG-3′; the FAST-1 binding element (FBE) competitor was 5′-CTG CCC TAA AAT GTG TAT TCC TAG GAA ATG 3′. The double-strand oligonucleotides used for gel mobility shift assay were obtained by annealing each oligonucleotide with its complementary antisense strand.

Oligo I–II, Oligo II, or Oligo I, with and without mutations, were also subcloned into a pGL3 promoter-luciferase reporter gene vector containing the SV40 minimal promoter (Promega, Madison, WI, USA).

To construct the human 90K minimal promoter-luciferase chimera (pGL3_hu90K) the fragment spanning from −115 to +42 of the human 90K 5′-flanking region (sequence number based on defining transcriptional start site as +1) was generated by PCR using the full length 90K promoter present in the pBluescript II KS (+) vector (a gift of Prof. Axel Ullrich, Max Plank Institutes, Munich, Germany)^21^ as template, the sequence 5′-CCG CTC GAG TGG GGA GTA TCA GCA GCA G-3′ as forward primer, and the sequence 5′-TAC CAA CAG TAC CGG AAT GC-3′ as reverse primer. The fragment was subcloned in pGL3 basic vector (Promega, Madison, WI, USA) at the *Xho*I and *Hin*dIII insertion sites.

### Transient expression

Transient transfections in FRTL-5 cells used a DEAE procedure^22^ and were performed as described^20^. Transfections used 2 μg of the pGL3 basic luciferase reporter gene or equivalent molar amounts of pGL3 containing the P2 rat 90K constructs with or without IRE mutation (P2IREM). pSVGH, 5 μg, was added to measure transfection efficiency. In some experiments, cotransfections were performed using 3 μg of pUSF1, pUSF2, or the control vector pCMV-tag2A. In other experiments, the same procedure was used to transfect cells with 2 μg of the SV40-driven pGL3 luciferase reporter vector or equivalent molar amounts of this vector containing Oligo I–II, Oligo II, or Oligo I with or without the above-indicated mutations. After transfection, cells were maintained 3–6 h in 6H medium and then shifted to 5H medium with no TSH. After 24 h in the 5H medium, cells were treated with TSH (1 × 10^−10^M), γ-IFN (100 U/ml), or TGF-β1 (5 ng/ml). Luciferase activity was assayed after 24 h. Values were normalized for total cell protein and growth hormone activity. The activity of the empty vector was considered as the control value unless otherwise noted.

Transfections of pGL3_hu90K were performed in U2OS cells using Lipofectamine 2000 reagent (ThermoFisher Scientific, Waltham, Massachusetts, USA). Briefly, 10,000 cells were co-transfected with the pGL3_hu90K or the empty vector (150 ng), in the presence or not of pUSF1 or pUSF2 vector (150 ng) and the *Renilla* luciferase reporter vector pRLTK (15 ng) (Promega, Madison, WI, USA). After 24 h, firefly luciferase activity was measured and normalized to that of *Renilla* using the Dual-Luciferase Reporter Assay System according to the manufacturer’s instructions (Promega, Madison, WI, USA).

### Nuclear extracts

To prepare nuclear extracts, FRTL-5 cells were grown in a complete 6H medium until 50–70% confluent. Cells were shifted in 5H medium with no TSH for 5 days and then stimulated with γ-IFN (100 U/ml), TSH (1 × 10^−10^ M), TGF-β1 (5 ng/ml), or combinations thereof for the times noted, usually 24 h. Nuclear extracts were prepared as described^20^. Protein concentration was determined by Bradford’s method (Bio-rad, Hercules, CA, USA) with crystalline bovine serum albumin (BSA) as standard. The extracts were aliquoted and stored at −70 °C.

### Electrophoretic mobility shift assay (EMSA)

DNA probes were the oligonucleotides described above. They were 5′ labeled with [γ-^32^P]ATP using T4 polynucleotide kinase (New England Biolabs). Binding reactions, 20 μl in volume, included 5 μg nuclear extract, 2% glycerol, 50 ng/ml polydI-dC, and 2 mM DTT in Binding Buffer: 20 mM HEPES, 0.1% NP40, 5 mM MgCl_2_, and 50 mM KCl. Where indicated, specific antisera (0.4 ng) or unlabeled oligonucleotide competitors (20× or 50× the concentration of labeled probe) were added for 15 min at room temperature. Reactions were initiated by adding 50,000 cpm labeled DNA probe; after 15 min at room temperature, samples were electrophoresed at 160 V on 5% native polyacrylamide gels in 1×TBE at room temperature. Gels were dried and autoradiography performed.

### USF1 production in *Escherichia coli*

Recombinant USF1 was produced using the pET system (Merck, Darmstadt, Germany). The USF1 cDNA was subcloned into the *Eco*RI site of the expression vector pET-30(+), allowing the His-Tag sequence to be linked to its N terminus. The protein was produced in *E. coli* BL21 (DE3) after a 4-h stimulation with 1 mM isopropyl-β-d-thiogalactopyranoside (IPTG) and purified according to the manufacturer’s procedures under reducing conditions. Purified USF1 was dialyzed against 20 mM HEPES, 0.1% NP40, 5 mM MgCl_2_, 50 mM KCl, 2% glycerol, 2 mM DTT, and concentrated in a Centricon 10 (Amicon, Beverly, MA, USA) to be used in EMSA. The purity and integrity of the recombinant protein were verified by SDS/PAGE.

### Analysis of public platforms for ChIP-seq and clinical data

To confirm the binding of USFs in the promoter region of the human 90K gene, we analyzed the publicly available ChIP-seq (chromatin immunoprecipitation followed by high-throughput sequencing) database generated by the ENCODE consortium, freely accessible at https://www.encodeproject.org/. The ChiIP-seq experiments were carried out on the human embryonic cell H1.

The cBioPortal platform, publicly available at https://www.cbioportal.org/, was analyzed to correlate 90K (both mRNA and protein) with TGF-β1 and USFs expression and to investigate the relationship between 90K levels and overall survival (OS) in patients affected by different types of cancer.

### Statistical significance

All experiments were repeated at least three times with different batches of cells. Values were the mean ± SD of these experiments where noted. Significance between experimental values was determined by a two-tailed Student’s *t*-test or two-way analysis of variance and was significant if *p* values were <0.05 when data from all experiments were considered.

### Materials

Highly purified bovine TSH was obtained from the hormone distribution program of the National Institute of Diabetes and Digestive and Kidney Diseases, National Institutes of Health (NIDDK-bTSH I-1; 30 U/mg). Rat γ-IFN was from Amgen (Thousand Oaks, CA, USA); recombinant IGF-I was from the Fujisawa Pharmaceutical Co. (Chūō, Tokyo, Japan). Antibodies against USF1 or USF2 were from Santa Cruz Biotechnology, Inc. (Dallas, TX, USA). Human platelet TGF-β1 was from Merck (Darmstadt, Germany). [γ-^32^P]ATP (3000 Ci/mmol) was from Amersham (Arlington Heights, IL, USA). The source of other materials was Merck (Darmstadt, Germany) unless otherwise noted.

## Results

### TGF-β1-increased 90K RNA levels and protein secretion

**Fig. 1:**
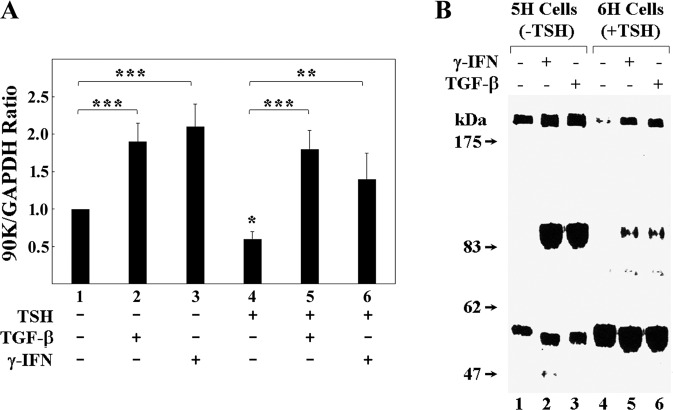
Effect of TGF-β1 on rat 90K mRNA and protein secretion in FRTL-5 cells. In **A**, RT-qPCR analyses to characterize the effect of TGF-β1 and γ-IFN on 90K mRNA expression in the presence or absence of TSH. In **B**, autoradiography of biolabeled secreted proteins immunoprecipitated by specific anti-90K serum. A single asterisk (*) denotes a significant decrease (*p* < 0.05); two asterisks (**) or three asterisks (***) denote a significant increase (*p* < 0.01 and *p* < 0.001, respectively). Data represent the mean ± SD of duplicate values determined in three separate experiments performed on different batches of cells.

Although FRTL-5 cells maintained in medium without TSH (5H medium) had a considerable expression of 90K mRNA detectable by RT-qPCR (Fig. 1A, bar 1), the addition of 5 ng/ml of TGF-β1 to the medium caused a significant increase in 90K mRNA levels (Fig. 1A, bar 2 vs 1). When 1 × 10^−10^M TSH was added to the 5H medium, there was, in contrast, a significant decrease in 90K mRNA levels (Fig. 1A, bar 4 vs 1), as previously reported^14^. The addition of TGF-β1 overcomes the suppressive action of TSH, increasing 90K RNA levels to the same extent of that observed when TGF-β1 was added to 5H medium (Fig. 1A, bar 5 vs 2).

The increase in 90K mRNA induced by TGF-β1 was as prominent as that induced by γ-IFN (Fig. 1A, bar 3 vs 1 and bar 6 vs 4), particularly in 6H cells because TSH, in the presence of insulin, reduces the γ-IFN effect, as previously reported^14^. RT-qPCR data were confirmed by northern blot analysis (see Fig. 1SA, B in Supplementary Materials).

Autoradiography of [^35^S]methionine-labeled proteins secreted by FRTL-5 cells and immunoprecipitated with a specific anti-90K antibody revealed the presence of a high molecular weight form of 90K (~200 kDa) and one of ~57 kDa, when cells were grown in 5H medium (Fig. 1C, lane 1). High molecular weight dimers of 90K protein have been identified by western blotting in studies on human fluids^11^ and the mechanisms involved in dimerization or oligomerization have been detailed^23^. The 200- and 57-kDa bands are likely corresponding to 90K dimers and protein degradation products, respectively. In some experiments, in 5H medium, a tiny band at ~90 kDa was also appreciable, corresponding to the 90K monomer^14^.

Cells incubated with γ-IFN or TGF-β1 for 24 h (Fig. 1C, lane 2 and 3, respectively) secreted a prominent ~90 kDa form, as well as an increased amount of the high molecular weight 200 kDa form of the 90K protein. The ~57 kDa form appeared increased, albeit slightly decreased in size. Moreover, an additional 47 kDa form appeared in the medium from γ-IFN-treated cells (Fig. 1C, lane 2). The increase in 90K secretion (monomer and dimer) was consistent with the effect of TGF-β1, as well as γ-IFN, to increase mRNA levels and protein synthesis, while that of 57- and 47-kDa forms was consistent with increased degradation of 90K in association with increased synthesis and secretion.

The addition of TSH to cells in a medium containing insulin (6H vs 5H), significantly decreased secretion of the ~200 kDa form of the protein and increased the amounts of lower molecular weight forms (Fig. 1C, lanes 4 to 6). This was consistent with increased degradation of 90K in the presence of TSH, whether synthesis was increased by TGF-β1 or γ-IFN. Thus, TSH does not affect TGF-β1-induced 90K RNA levels but increases 90K protein degradation.

### Identification of the TGF-β1 responsive element in the minimal 90K functional promoter

**Fig. 2:**
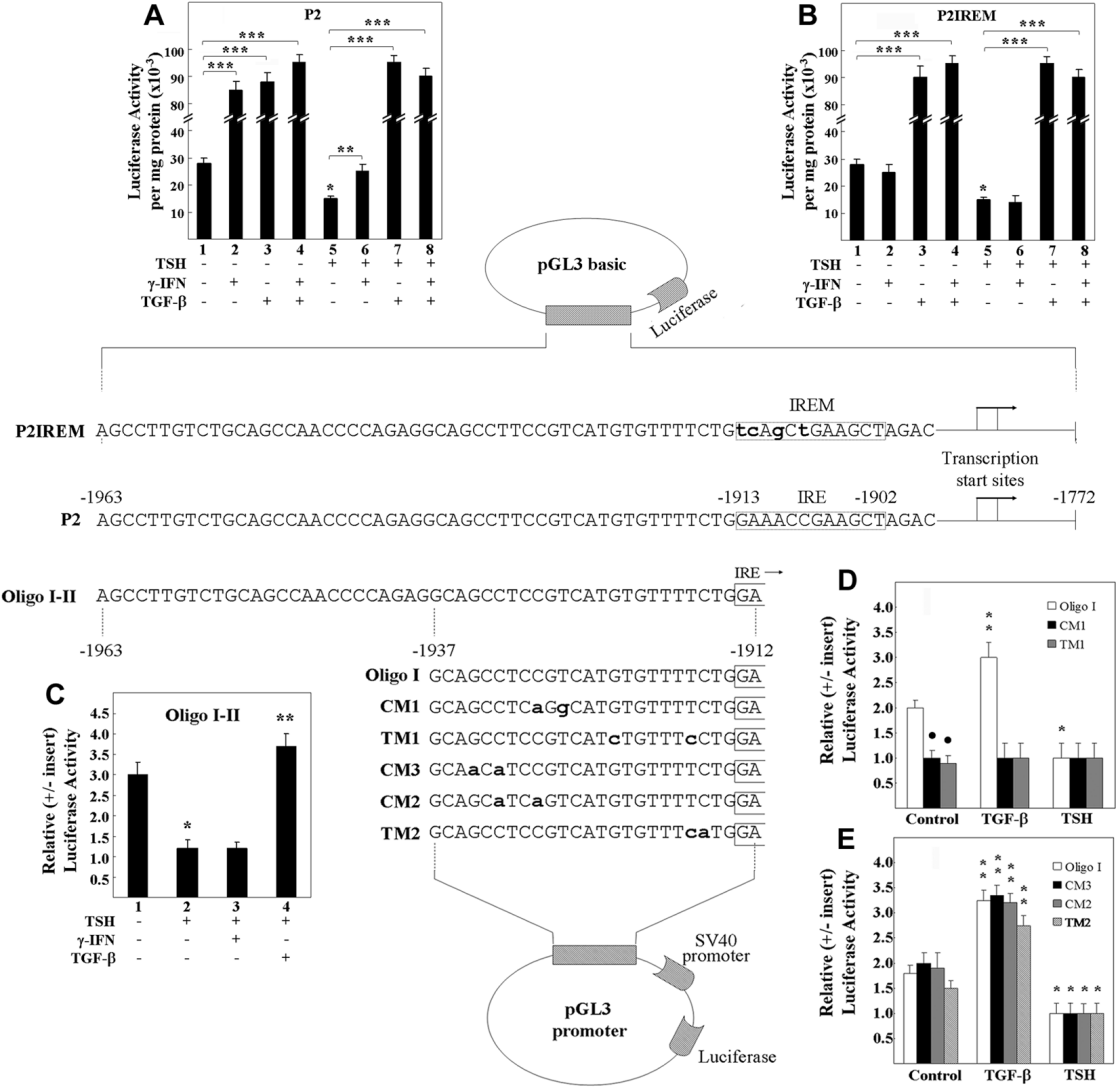
Ability of TGF-β1 to increase promoter activity of rat 90K in FRTL-5 cells. In the upper diagram, the sequence of the rat 90K minimal promoter, P2 fragment, or the same construct with 4 bp mutations in the interferon response element (IRE), P2IREM, are depicted as inserted in a pGL3 basic luciferase reporter vector. The IRE is boxed to show the mutated nucleotides (lowercase letters). Luciferase activity was normalized for protein and growth hormone activity. In **A**, luciferase activity of the P2 construct. In **B**, luciferase activity of the P2IREM construct. In the lower diagram, the region between −1963 and −1912 bp upstream of the IRE, termed Oligo I–II, or the region between −1937 and −1912, termed Oligo I, or 5 different constructs with 2 bp mutations, were subcloned in a pGL3 promoter-luciferase reporter vector. Luciferase activity was expressed as relative to a control construct with no insert. In **C**, luciferase activity of Oligo I–II. In **D**, promoter activity of CM1 and TM1 is lost compared to Oligo I. In **E**, promoter activity of CM3, CM2 and TM2 is similar to that of Oligo I. A single asterisk (*) denotes a significant decrease in promoter activity (*p* < 0.05); two asterisks (**) or three asterisks (***) denote a significant increase in promoter activity (*p* < 0.05 and *p* < 0.01, respectively), a dark circle (●) denotes a significant decrease in constitutive promoter activity (*p* < 0.05).

We measured the activity of the rat 90K minimal promoter by luciferase assay as described previously^20^, using the construct containing the fragment spanning position −1963 to −1772 bp of the 90K 5′-flanking region (P2 construct) (Fig. 2, middle diagram). After transfection, FRTL-5 cells were maintained in 5H medium and stimulated with 5 ng/ml TGF-β1, 100 U/ml γ-IFN, or 1 × 10^−10^ M TSH (Fig. 2A). We observed that the luciferase activity of the construct in response to TGF-β1 or γ-IFN mimicked the effects of both on RNA levels (Fig. 2A vs Fig. 1B). Based on the activity of the P2 construct, we concluded that TGF-β1 acted on the same minimal promoter that was sensitive to γ-IFN and TSH. This region includes the IRE and corresponds to the minimal promoter defined by sequence homology between rat, human, and mouse genes^20^.

The insertion of 4 bp mutations in the IRE of the P2-luciferase construct (P2IREM) (Fig. 2, upper diagram) abolished the ability of γ-IFN to increase promoter activity (Fig. 2B, bar 2 and 6), but did not influence significantly the effect of TGF-β1 or TSH (Fig. 2B, bar 7 and 5, respectively), indicating that TGF-β1 activity was not mediated by the IRE.

The sequence of P2 upstream of the IRE, termed Oligo I–II, was inserted into a pGL3 vector with a SV40 promoter (Fig. 2, lower diagram). In 5H medium, the Oligo I–II construct had a significant constitutive activity compared with its control vector without the insert (Fig. 2C, bar 1). TSH significantly decreased constitutive activity (Fig. 2C, bar 2). TGF-β1, but not γ-IFN, overcame the TSH-induced decrease in promoter activity (Fig. 2C, bar 4 vs 3). The Oligo I–II region was, therefore, the TSH and TGF-β1 site of action.

By inserting Oligo I wild type (−1937 to −1912) or Oligo I with 2 bp mutations into a pGL3 promoter, we had previously determined the site for constitutive 90K expression and TSH suppression^20^. Similarly, we could localize the TGF-β1 action within this region. Coordinate results were obtained when vector constitutive activity was evaluated simultaneously for TGF-β1- or TSH-responsiveness. In particular, as was the case for TSH-suppressive activity, the increased response to TGF-β1 was present in Oligo I construct, but was lost, along with constitutive activity, in the constructs containing mutations between −1929 and −1917 bp (CM1 and TM1) (Fig. 2D). The promoter activity exhibited by Oligo I and CM2, CM3, TM2 mutants were similar, as was their TGF-β1 and TSH response (Fig. 2E). Since mutant CM1 lost both activities as did TM1, but not TM2, CM2, nor CM3, it was evident that the critical residues for constitutive activity and for both TGF-β1 or TSH activity involved the sequence spanning −1929 to −1917 bp, and did not involve the 5′ nucleotide mutation in CM1 (C-1929A) and the 3′ nucleotide mutation in TM1 (T-1917C).

We preliminarily interpreted these data as follows. An element important for the constitutive activity of the rat 90K minimal promoter existed between −1929 and −1917 bp. This same element was also important for both TGF-β1-increased and TSH-decreased 90K activity. EMSA data below support this interpretation and define the *cis* element as an E-box and the *trans* factors involved as USF1 and USF2.

### An E-box and USF transcription factors regulate constitutive and TGF-β1-induced expression of rat 90K

**Fig. 3:**
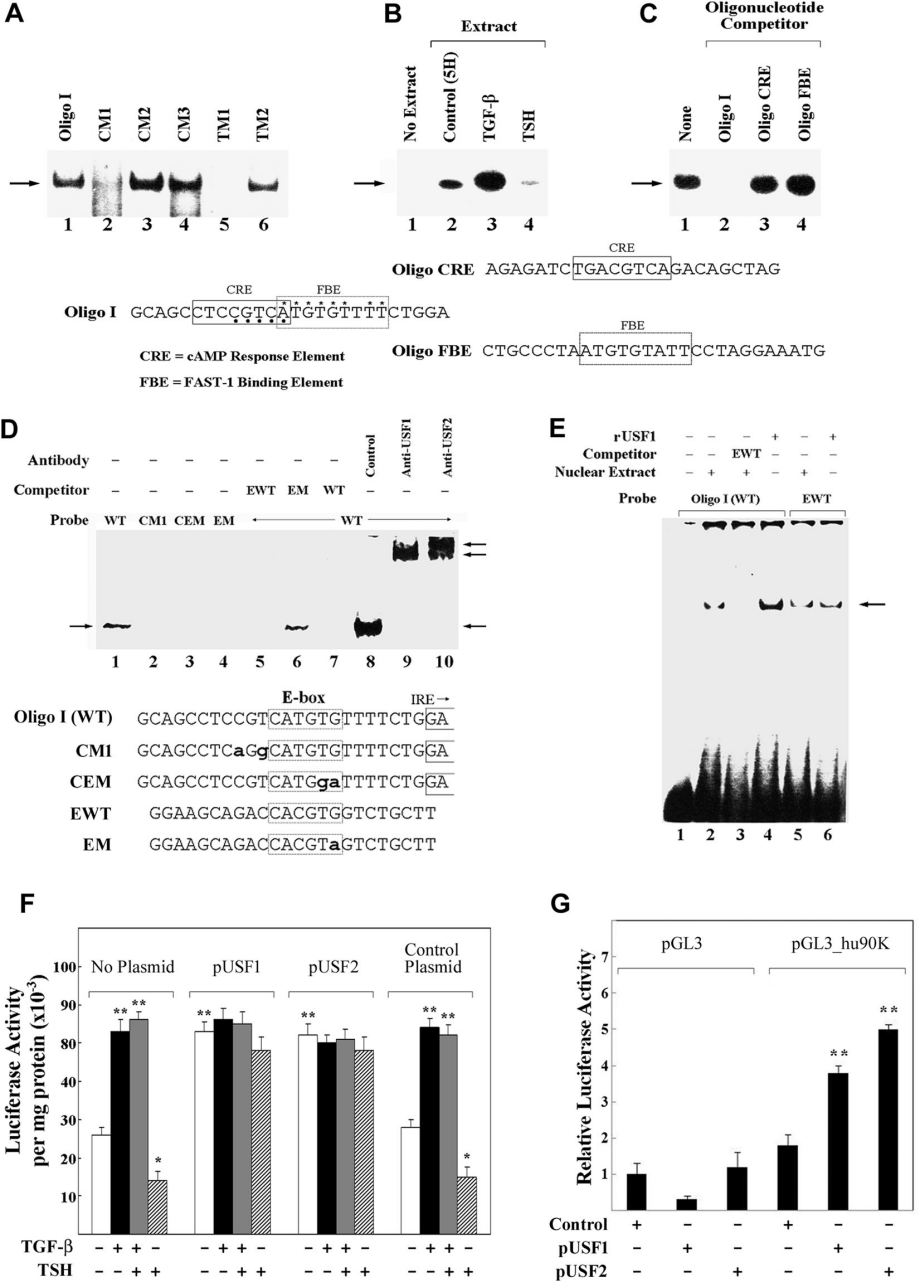
Identification of a specific complex binding to the rat 90K minimal promoter important for TGF-β1-induced or TSH-suppressed expression of the 90K gene. In **A**, EMSA of nuclear extracts from cells maintained in 5H medium (no TSH) using as probes the radiolabeled double-strand oligonucleotides with the sequence of Oligo I or the ones with 2 bp substitutions reported in the lower diagram of Fig. 2. In **B**, EMSA of nuclear extracts from cells treated with TGF-β1 or TSH and incubated with Oligo I as a probe. In **C**, EMSA of nuclear extracts from cells treated with TGF-β1 using Oligo I as probe and each of the double-strand oligonucleotides depicted in the bottom as competitor of complex formation, i.e., Oligo I, with the potential CRE or FAST-1 (FBE) binding sites boxed, Oligo CRE, an unrelated oligonucleotide containing the consensus sequence for CRE, and Oligo FBE, an unrelated oligonucleotide with the consensus sequence for FBE. Nucleotides in Oligo I that are the same as the CRE are noted with dark circles; nucleotides in Oligo I that are the same as the FBE are noted with asterisks, In **D**, EMSA of nuclear extracts from TGF-β1-treated cells incubated with the indicated probes, competitors (20× concentration over labeled probe) or antisera (control, anti-USF1 or anti-USF2). CEM is Oligo I with 2 bp mutations in the E-box; EWT is an oligonucleotide with a consensus E-box but no other sequence homology to Oligo I; EM is an unrelated oligonucleotide with a mutation in the E-box. The lowest arrow on the right denotes the native complex; the two upper arrows denote the supershifted complexes. In **E**, EMSA of nuclear extracts from TGF-β1-treated cells incubated with Oligo I or EWT used as probes. In lanes 4 and 6, they were respectively incubated with recombinant USF1 (rUSF1). In **F**, rat FRTL-5 cells were co-transfected with the pGL3 vector containing the P2 construct of the rat 90K gene and the plasmid containing the full-length cDNAs encoding USF1 (pUSF1) or USF2 (pUSF2) or the control vector pCMV-tag2A (control plasmid) or nothing (no plasmid). pSVGH was added to measure transfection efficiency. Values were normalized for cell protein and growth hormone activity. In **G**, human U2OS cells were co-transfected with pGL3 control vector or the pGL3 vector containing the human 90K minimal promoter (pGL3_hu90K), plus the Renilla luciferase reporter vector pRLTK to measure transfection efficiency, and with pUSF1 or pUSF2 or control vector as in **F**. Values were normalized for cell protein and Renilla activity, and expressed as relative to pGL3 control vector with no insert. A single asterisk (*) denotes a significant TSH-induced decrease in constitutive promoter activity (*p* < 0.05); two asterisks (**) denote a significant increase in promoter activity induced by TGF-β1 or by transfection with pUSF1 or pUSF2 (*p* < 0.01).

As previously reported^20^, we performed EMSA to evaluate the effects of the CM1, CM2, CM3, TM1, and TM2 mutations on *trans* factor binding to the Oligo I region defined functionally as important for constitutive as well as TGF-β1-enhanced or TSH-suppressed 90K activity (Fig. 3A). A prominent protein/DNA complex was noted incubating radiolabeled Oligo I with nuclear extracts of cells maintained in 5H medium (Fig. 3A, lane 1). This complex was nearly eliminated in incubations with radiolabeled CM1 or TM1 oligonucleotides (Fig. 3A, lanes 2 and 5, respectively) but was unaltered using radiolabeled CM3 or CM2 oligonucleotides (Fig. 3A, lanes 3 and 4). The complex was only partly attenuated in incubations with radiolabeled TM2 oligonucleotide (Fig. 3A, lane 6).

More importantly, formation of the complex was increased in extracts from cells treated with 5 ng/ml TGF-β1 (Fig. 3B, lane 3 vs 2), but decreased in extracts from cells treated with TSH (Fig. 3B, lane 4 vs 2).

The sum of these data suggested that the presence of this complex was important for constitutive 90K gene expression, was increased by TGF-β1, but was decreased by TSH.

In reviewing the sequence of Oligo I, we noted a potential cyclic AMP responsive element (CRE) but found no inhibition of the complex by an oligonucleotide with a consensus CRE (Fig. 3C, lane 3). Similarly, we noted a potential FAST-1 binding site but found no inhibition of the complex by an oligonucleotide with a consensus FAST-1 binding element (FBE) (Fig. 3C, lane 4). The exclusion of FAST-1 as a potential binding site was important given the extent of homology and given that FAST-1 is involved in TGF-β1 signaling^1^.

We noted, however, a potential E-Box CANNTG at −1926 to −1921 bp, and could show that binding of the constitutive complex was not duplicated by an Oligo I with a critical mutation in the potential E-box (CEM), CATG**ga** (mutation in bold and small letters), as was the case for the CM1 oligonucleotide (Fig. 3D, lanes 3 and 2, respectively, vs lane 1). Formation of a complex with the same mobility was obtained with a radiolabeled oligonucleotide with a consensus E-box (CACGTG) and no other sequence homology to Oligo I (EWT) (see Fig. 3E, lines 5 and 6), but did not with the same oligonucleotide carrying the E-box mutation CACG**ga** (EM) (Fig. 3D, lane 4). When used as competitors, both oligonucleotides, Oligo I and EWT, were inhibitory at the same concentrations (Fig. 3D, lanes 5 and 7 vs 1). In contrast, there was no inhibition when EM was used as competitor (Fig. 3D, lane 6). These data supported the conclusion that the element important for constitutive as well as TGF-β1-increased 90K activity of the rat 90K minimal promoter was an E-box with the sequence CATGTG, consistent with the known E-box site CANNTG.

The E-Box is known to bind the basic helix-loop-helix zipper proteins, upstream stimulatory factors 1 and 2 (USF1 and USF2)^24^. When antisera to USF1 and USF2, but not a control serum, were included in the assay, the native complex was eliminated (low arrow) and supershifted complexes appeared (Fig. 3D, lanes 9 and 10 vs 8). The increase in density of the complex, independent of the sera added, was presumed to be a nonspecific salt effect of the antisera buffer (Fig. 3D, lanes 8 to 10). Anti-USF2 generated two supershifted complexes, the upper of which was most prominent (Fig. 3D, lane 10, arrows). Anti-USF1 appeared to generate a major supershifted complex with the same mobility as the faster migrating one supershifted by anti-USF2, and a smaller amount of the upper complex (Fig. 3D, lane 9, arrows).

The ability of anti-USF1 and anti-USF2 to generate a supershifted complex with the same mobility and to completely eliminate the native complex suggested that the latter was represented by a USF1/USF2 heterodimer. The differences in the pattern of supershifted complexes obtained with anti-USF1 and anti-USF2 suggested, however, that USF homodimers might also be interacting with the E-box of the 90K gene in extracts from TGF-β1-treated cells.

Using Oligo I or the unrelated EWT oligonucleotide (Fig. 3E, EWT) as radiolabeled probes, we found complexes with identical mobility in EMSA with nuclear extracts from TGF-β1-treated cells (Fig. 3E, lanes 2 and 5). The same-sized complexes were also formed with recombinant USF1 (Fig. 3E, lanes 4 and 6). More importantly, the addition of full-length pUSF1 or pUSF2 to transfections enhanced the constitutive expression of P2, eliminated the TGF-β1-induced increase, and nearly completely attenuated the TSH-induced suppression of constitutive P2 expression (Fig. 3F). This was not duplicated by the control plasmid.

Given the high sequence homology between the rat and human 90K minimal promoters, including the same located IRE and E-box^20^, we would verify if USFs were able to induce the human 90K gene. Co-transfection of U2OS cells with pUSF1 or pUSF2 expression plasmids in the presence of pGL3_hu90K significantly enhanced the luciferase activity of the reporter vector (Fig. 3G), indicating a direct effect of USF in inducing human 90K gene expression.

### USFs bind the E-box in the promoter region of the human 90K gene

**Fig. 4:**
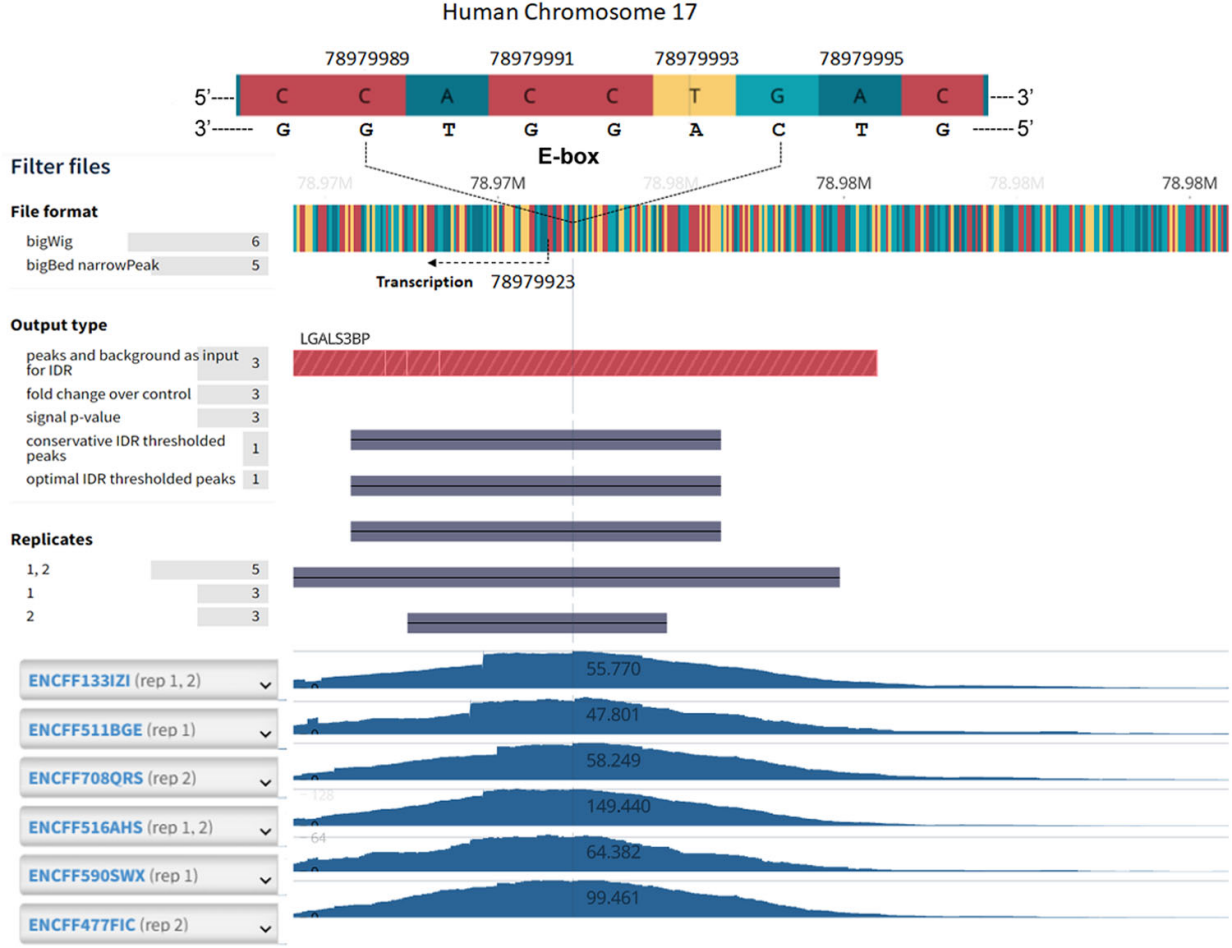
Genomic ChIP-seq reveals that USFs bind the E-box present in the promoter region of the human 90K (LGALS3BP) gene. ChIP-seq analysis was performed in the publicly available ENCODE platform. Search parameters were the human embryonic cells H1, USF1 as a transcription factor, and LGALS3BP as the target gene. Six different experiments showed a peak region in the LGAL3BP promoter in Chromosome 17, automatically aligned. The platform provided also the number of nucleotide position in the chromosome. The highest point of the peaks overlapped with the consensus motif CANNTG binding site for USF, depicted on the top. The arrow indicates the direction of gene transcription.

The ability of USFs to bind the E-box present in the human 90K gene promoter was confirmed by analyzing the ChIP-seq database generated by the ENCODE consortium. By selecting the human embryonic cell H1 as Biosample and browsing the platform for USF1, as a transcription factor, and human 90K (LGALS3BP), as the target gene, a peak region in the LGALS3BP promoter sequence of Chromosome 17 was identified in six different experiments (Fig. 4, lower diagram). As expected, the highest point of the peaks overlapped with the consensus motif CANNTG binding site for USF (Fig. 4, upper diagram).

### Human 90K (LGALS3BP) levels directly correlate with TGF-β1 and USFs expression and affect cancer patient prognosis

**Fig. 5:**
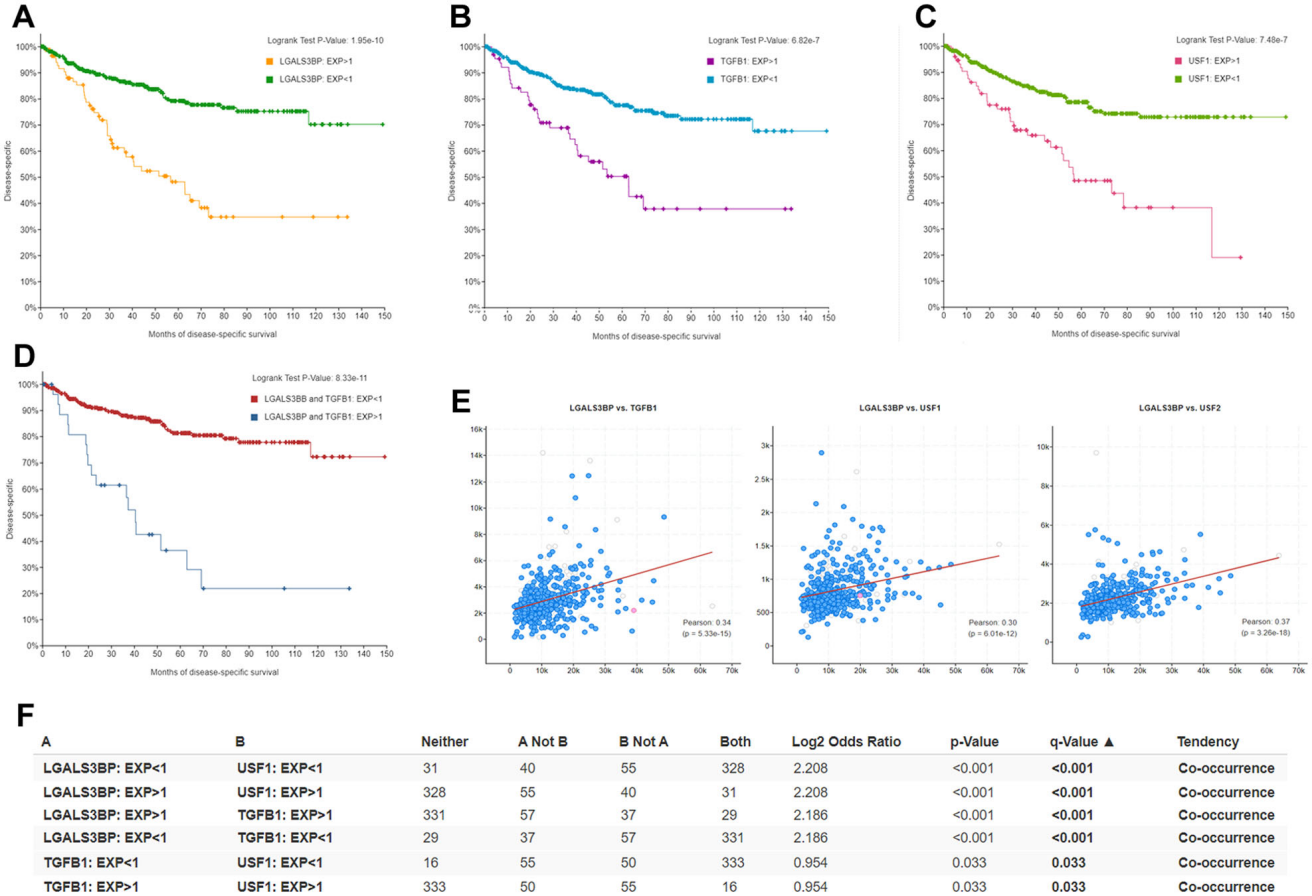
Human 90K (LGALS3BP) expression is associated with poor prognosis and directly correlates with TGF-β1 and USFs. Using the public cancer database cBioPortal, the TCGA PanCancer Atlas cohort of patients affected by clear cell renal cell carcinoma (ccRCC) was analyzed (*N* = 512). In **A**, Kaplan–Meier curves of disease-specific OS stratified by high (EXP > 1) or low (EXP < 1) expression of LGALS3BP. In **B**, patients were stratified by TGF-β1 expression; in **C**, by USF1 expression; and in **D**, by concomitant LGALS3BP/TGF-β1 high or low expression. In **E**, scatterplot correlation between LGALS3BP and TGF-β1 (left), LGALS3BP and USF1 (middle); LGALS3BP and USF2 (right), as visualized by cBioPortal. In **F**, table elaborated by cBioPortal showing tendency of co-occurrence among 90K, TGF-β1, and USF1.

The analysis of different cancer data sets included in the public cBioPortal platform revealed a general association between high tumor levels of LGALS3BP (both mRNA and protein) and shorter survival in patients affected by different types of cancer. The effect reached statistical significance in some tumors such as breast cancer, colorectal cancer, and clear cell renal cell carcinoma (ccRCC). The latter neoplasm showed the highest statistical power in distinguishing prognosis between patients with high and low LGALS3BP levels. In particular, in a cohort of 512 patients with ccRCC included in the TCGA PanCancer Atlas database, the median disease-specific OS was significantly shorter in patients with high LGALS3BP (EXP > 1) compared to those with low LGALS3BP (EXP < 1) (56.68 months vs not reached, *p* < 0.001) (Fig. 5A). Similar results were observed among patients with high (EXP > 1) and low (EXP < 1) TGF-β1 (median disease-specific OS 53.42 vs 116.84 months, *p* < 0.001) (Fig. 5B), and among patients with high (EXP > 1) or low (EXP < 1) USF1 (median disease-specific OS 56.68 vs not reached, *p* < 0.001) (Fig. 5C). Importantly, the magnitude of the effect on survival was significantly increased when patients with concomitant high LGALS3BP and high TGF-β1 (HH) were compared with those with low LGALS3BP and low TGF-β1 (LL), indicating a synergistic effect between the two genes (Fig. 5D). Kaplan–Meier curves showed that the group HH had significantly shorter survival compared with the group LL (median disease-specific OS 40.44 months vs not reached, *p* < 0.001) (Fig. 5D). Importantly, in tumor samples, the expression levels of LGALS3BP showed a direct correlation with those of TGF-β1 (*R* = 0.34, *p* < 0.001), USF1 (*R* = 0.30, *p* < 0.001), and USF2 (*R* = 0.37, *p* < 0.001) (Fig. 5E). In addition, in cancer cells, there was a strong tendency of co-occurrence between high LGALS3BP, high USF1, high USF2, and high TGF-β1 expression (*p* < 0.001) or low LGALS3BP, low USF1, low USF2, and low TGF-β1 expression (*p* < 0.001) (Fig. 5F). Overall, clinical data support a direct relationship between the activation of the TGF-β1/USFs/90K axis and poor prognosis.

## Discussion

In this study, we showed that TGF-β1 increases 90K gene expression by increasing USF1 and USF2 binding to its promoter, in the same E-box involved in the hormonal regulation of the gene.

In fact, we previously demonstrated that USF1 and USF2 were the relevant transcription factors controlling constitutive 90K expression and that TSH was able to decrease 90K expression by reducing USFs binding to the E-box^20^.

Taken together, this and our previous report suggest that 90K gene expression depends on the interaction of the E-box located in its promoter with USF1 and USF2, and that this interaction is regulated by TGF-β1 and TSH: TGF-β1 increments USF binding to the E-box and increases 90K gene expression, while TSH reduces USF binding to the E-box and decreases 90K gene expression.

A role of USFs in the TGF-β1 signaling has been already suggested in studies on the plasminogen activator inhibitor-1 (PAI-1) gene^25–28^, also known as SERPINE1, but so far no other TGF-β-induced gene has been reported to be dependent on USF activity. The present report unequivocally, therefore, establishes the importance of USF family transcription factors in the TGF-β1 signaling.

Classically, TGF-β1 signaling is mediated by receptor-associated Smad2 and Smad3 proteins which form complexes with Smad4 and accumulate in the nucleus where regulates gene expression^29,30^. Smad2, Smad3, or Smad4 can mediate TGF-β1-induced transcription by binding directly to DNA in specific Smad Binding Element (SBE)^31,32^. No SBE site exists in 90K minimal promoter, but we cannot exclude an interaction of USFs with Smads to act cooperatively elsewhere in the intact 90K gene. Smads can cooperate with other transcription factors and/or can interact with co-activator such as FAST-1 to activate transcription^33,34^. Since a potential FAST-1 consensus sequence was present in 90K minimal promoter, we specifically excluded FAST-1 as a mediator of the TGF-β1 action on 90K gene. The role of the Smad system in the TGF-β1 regulation of 90K gene described herein remains to be defined, even though Smad-independent pathways have been described^35,36^.

The effect of TGF-β1 to increase 90K gene expression is quite surprisingly given that TGF-β1 is a suppressor of the immune system^2,3^ and decreases MHC class I expression in FRTL-5^15^, while 90K is an immune-stimulator and increases MHC class I. The finding that USFs are directly involved in the TGF-β1 regulation of 90K gene provides a mechanistic explanation: TGF-β1 suppresses MHC class I while increasing 90K because different transcription factors are involved in the regulation of the two genes. In fact, in FRTL-5 cells, TGF-β1 has been shown to decrease MHC class I gene expression by regulating diverse *trans* factor interactions with two elements located within the “hormonal regulatory region” of its promoter (−203 to −90 bp)^15^. The first element, called Enhancer A (−180 to −170 bp), can interact with either an inducer factor, the heterodimer NF-kB p50/fra-2 (MOD-1), or an inhibitor factor, the heterodimer NF-kB p50/p65. TGF-β1 reduces the interaction of MOD-1 with Enhancer A while increasing that of NF-kB p50/p65. Both, reduced MOD-1 and increased p50/p65, suppress class I expression^15^. The second element, called downstream regulatory element (DRE) (−127 to −90 bp) is the binding site of the inhibitor factor TSEP-1, a ubiquitously-expressed Y-box protein. TGF-β1 increases the binding of TSEP-1 to DRE and, as a consequence, suppresses class I expression^15^. These interactions appear to counteract or dominate the inductive effect of 90K to increase class I.

In addition, a functional explanation can be provided for the effect of TGF-β1 on MHC class I and 90K: the upregulation of 90K expression is not related to the immune response but may reflect the TGF-β1-induced cellular microenvironment modifications that facilitate cancer progression. In fact, experimental and clinical data indicate that 90K is implicated in tumor invasion and metastases, probably as a result of its ability to promote cell-to-cell and cell-to-extracellular matrix (ECM) adhesion^9,37,38^. Consistently, high expression levels of 90K, both in serum or in tumor cell, have been observed in patients with different types of cancer and were associated with a higher incidence of metastasis and poor prognosis^39–41^.

Similarly, TGF-β1 is increased in most human cancer, and high expression levels correlate with more advanced stages of malignancy and decreased survival^42–44^. The pro-metastatic effect of TGF-β1 is mostly related to its ability to remodel tumor microenvironment^45–48^ and to promote epithelial–mesenchymal transition (EMT)^49,50^.

We propose that in the context of the changing induced by TGF-β1 in the tumor microenvironment, the axis TGF-β1/USF/90K may take a part in the process of tumor progression. This possibility was further supported by our analysis of the cBioPortal database showing a significant increased mortality in patients affected by ccRCC with high levels of 90K expression in tumor samples. Similarly, high levels of TGF-β1 or USF1 were associated with a dismal prognosis. Moreover, a synergistic effect of 90K and TGF-β1 to predict higher mortality was observed. Importantly, in this cancer population, 90K levels directly correlated with TGF-β1 and USFs expression.

Notably, the other gene activated by TGF-β1 through USF is PAI-1, a critical factor in the tumor invasion program^51–57^ and implicated in the TGF-β1-induced EMT^58,59^.

The importance of TGF-β1 in the process of cancer invasion and diffusion has led to the development of pharmacological inhibitors of the TGF-β1 signaling, and currently, some agents are in the early stages of clinical trials^60^. PAI-1 has also been proposed as a target for cancer therapy^61^. Similarly, we would propose 90K as another possible anti-cancer target.

In conclusion, the results of the present study on the effect of TGF-β1 on 90K transcription and the role of USF1 and USF2 in the regulation of 90K gene expression have uncovered a novel possible role for 90K in the TGF-β1-driven tumor progression program. In this regard, 90K may become a suitable candidate for anti-cancer therapy.

## Supplementary information

Figure S1

Nothern Blot

Supplementary Figure Legends

Supplementary Results
