# Correction: Tgf-β1 transcriptionally promotes 90K expression: possible implications for cancer progression

**DOI:** 10.1038/s41420-021-00490-4

**Published:** 2021-05-11

**Authors:** Antonino Grassadonia, Vincenzo Graziano, Sara Pagotto, Angelo Veronese, Cesidio Giuliani, Marco Marchisio, Paola Lanuti, Michele De Tursi, Maurizia D’Egidio, Pietro Di Marino, Davide Brocco, Patrizia Vici, Laura De Lellis, Alessandro Cama, Clara Natoli, Nicola Tinari

**Affiliations:** 1 Department of Medical, Oral and Biotechnological Sciences and Center for Advanced Studies and Technology (CAST), G. D’Annunzio University, Chieti, Italy; 2 Cancer Research UK Cambridge Institute, University of Cambridge, Cambridge, UK; 3 Department of Medicine and Ageing Sciences and Center for Advanced Studies and Technology (CAST), G. D’Annunzio University, Chieti, Italy; 4 Division of Medical Oncology 2, IRCCS Regina Elena National Cancer Institute, Rome, Italy; 5 Department of Pharmacy and Center for Advanced Studies and Technology (CAST), G. D’Annunzio University, Chieti, Italy

**Keywords:** Growth factor signalling, Transcriptional regulatory elements

Correction to: *Cell Death Discovery*

10.1038/s41420-021-00469-1 published online 22 April 2021

The original version of this article unfortunately contained a mistake in one of the author names. The correct name is Pietro Di Marino. We apologise for the mistake. The original article has been corrected.

