# Supplementary figures and images for: Tgf-β1 transcriptionally promotes 90K expression: possible implications for cancer progression
Source: Cell Death Discov. 2021 Apr 22;7:86. doi: 10.1038/s41420-021-00469-1 (PMC8062489; doi:10.1038/s41420-021-00469-1)

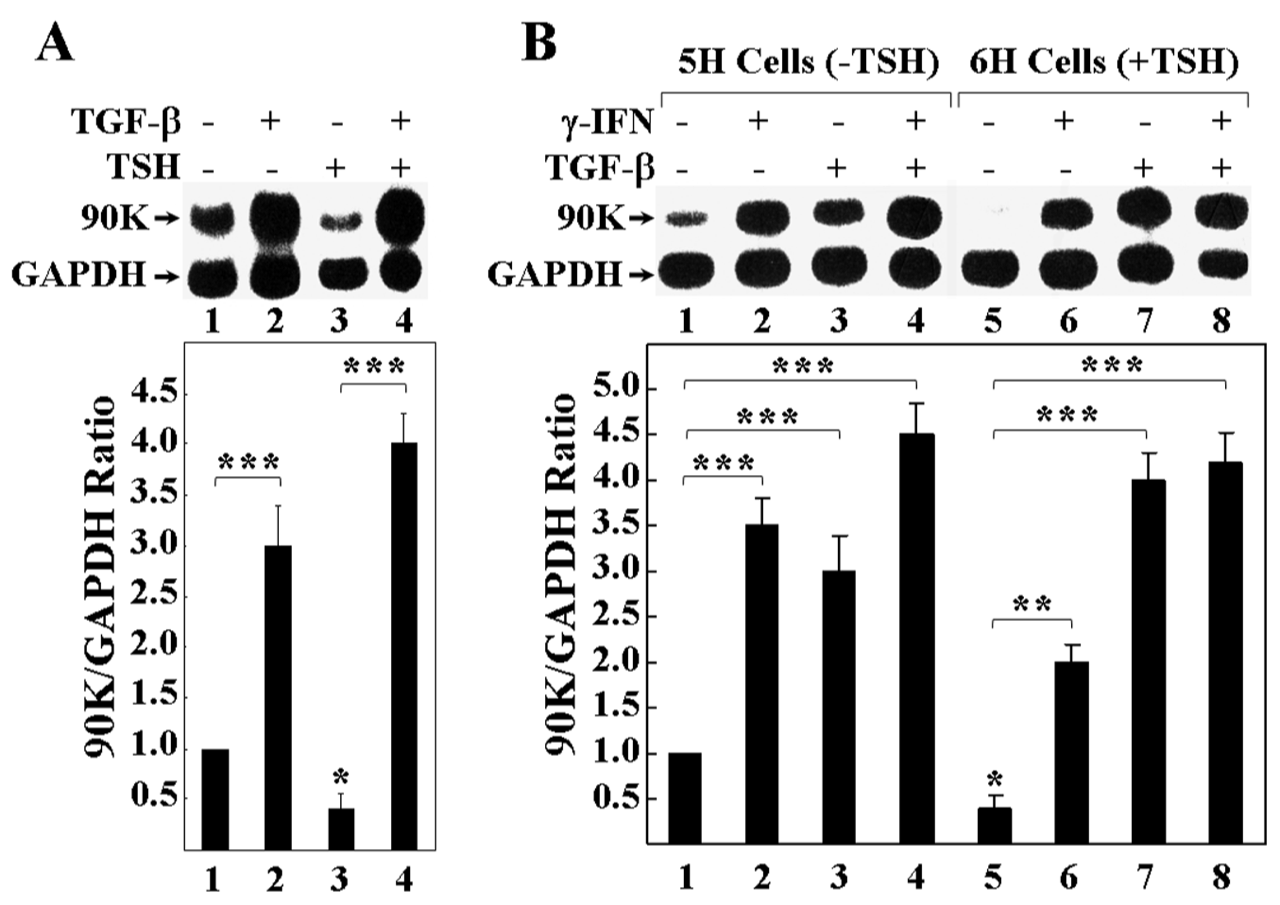

Supplement: Supplementary file 1 — Figure S1 [file 41420_2021_469_MOESM1_ESM.tif]
