# Supplementary material for: Tgf-β1 transcriptionally promotes 90K expression: possible implications for cancer progression
Source: Cell Death Discov. 2021 Apr 22;7:86. doi: 10.1038/s41420-021-00469-1 (PMC8062489; doi:10.1038/s41420-021-00469-1)
Supplement: Supplementary file 2 — Nothern Blot [file 41420_2021_469_MOESM2_ESM.pdf]

Ability of TGF-β1 to increase 90K mRNA levels

FRTL-5 cells maintained in medium without TSH (5H medium) had a considerable expression of 90K mRNA detectable by Northern analysis (Fig. 1S A, lane 1). The addition of 5 ng/ml of TGF-β1 to the medium caused a significant increase in 90K mRNA levels (Fig. 1S A, lane 2 *vs* 1). When $1 x 10^{-10}$M TSH was added to the 5H medium, there was, in contrast, a significant decrease in 90K mRNA levels (Fig. 1S A, lane 3 *vs* 1), as previously reported[14]. The addition of TGF-β1 overcomes the suppressive action of TSH, increasing 90K RNA levels to the same extent or to a slightly higher level than that observed when TGF-β1 was added to 5H medium (Fig. 1S A, lane 4 *vs* 2 and Fig. 1S B, lane 7 *vs* 3 in 6H *vs* 5H cells). The increase in 90K mRNA induced by TGF-β1 was as prominent as that induced by γ-IFN (Fig. 1S B, lane 2 *vs* 1 and lane 6 *vs* 5), particularly in 6H cells because TSH, in the presence of insulin, reduces the γ-IFN effect, as previously reported[14]. The effect of TGF-β1 was slightly additive to that of γ-IFN in 5H cells, while was equivalent in 6H cells (Fig. 1S B lanes 4 and 8), consistent with a decrease of γ-IFN effect, but not of TGF-β1, induced by TSH.

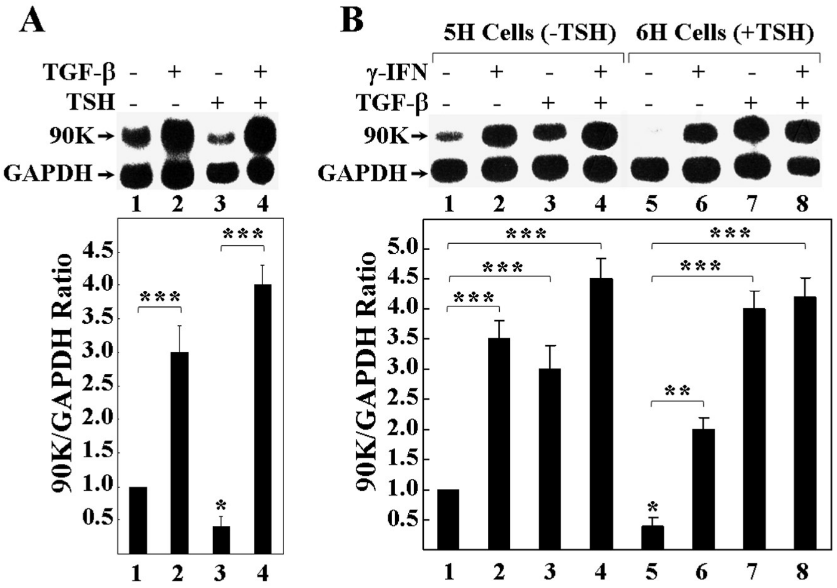

Figure 1S. Northern blot analyses of the effect of TGF-β1 on rat 90K mRNA in FRTL-5 cells. In (**A**), Northern analyses to characterize the effect of TGF-β1 in the presence or absence of TSH. In (**B**), the effect of TGF-β1 on 90K mRNA expression is compared with that of γ-IFN both in 5H cells (no TSH), and in 6H cells (with TSH). A single asterisk (*) denotes a significant decrease ($p<0.05$); two asterisks (**) or three asterisks (***) denote a significant increase ($p<0.05$ and $p<0.01$, respectively). Data represent the mean ± SD of duplicate values determined in 3 separate experiments performed on different batches of cells.
