# Supplementary material for: Tgf-β1 transcriptionally promotes 90K expression: possible implications for cancer progression
Source: Cell Death Discov. 2021 Apr 22;7:86. doi: 10.1038/s41420-021-00469-1 (PMC8062489; doi:10.1038/s41420-021-00469-1)
Supplement: Supplementary file 3 — Supplementary Figure Legends [file 41420_2021_469_MOESM3_ESM.docx]

**Figure 1S. Northern blot analyses of the effect of TGF-β1 on rat 90K mRNA in FRTL-5 cells.** In (**A**), Northern analyses to characterize the effect of TGF-β1 in the presence or absence of TSH. In (**B**), the effect of TGF-β1 on 90K mRNA expression is compared with that of γ-IFN both in 5H cells (no TSH), and in 6H cells (with TSH). A single asterisk (*) denotes a significant decrease (p<0.05); two asterisks (**) or three asterisks (***) denote a significant increase (p<0.05 and p<0.01, respectively). Data represent the mean ± SD of duplicate values determined in 3 separate experiments performed on different batches of cells.
